# Age Differences in Visual Attention and Responses to Intergenerational and Non-intergenerational Workplace Conflicts

**DOI:** 10.3389/fpsyg.2021.604717

**Published:** 2021-06-07

**Authors:** Dannii Y. Yeung, Derek M. Isaacowitz, Winnie W. Y. Lam, Jiawen Ye, Cyrus L. K. Leung

**Affiliations:** ^1^Psychology Laboratories, Department of Social and Behavioural Sciences, City University of Hong Kong, Kowloon, Hong Kong; ^2^Department of Psychology, Northeastern University, Boston, MA, United States; ^3^Department of Applied Psychology, Lingnan University, Tuen Mun, Hong Kong

**Keywords:** intergenerational workplace conflict, visual fixation, older workers, emotional reactions, conflict management

## Abstract

Intergenerational conflict occurs commonly in the workplace because of age-related differences in work attitudes and values. This study aimed to advance the current literature on aging and work by examining whether younger and older workers differ in their visual attention, emotional responses, and conflict strategies when observing hypothetical conflict vignettes involving a coworker from a similar or dissimilar age group. The indirect effect of age group on emotional responses and conflict strategies through visual fixation on conflict scenes was also examined. Utilizing eye tracking, the visual attention of younger and older workers while watching two hypothetical workplace task conflict videos was recorded and compared. The participants were also asked to imagine how they would respond if they were the main actor in the vignettes. A total of 94 working adults, including 48 younger workers and 46 older workers, participated in the eye tracking experiment. Older workers reported fewer negative and more positive emotions than their younger counterparts after watching the conflict videos, particularly those on the non-intergenerational conflict. Younger workers used more dominating in the intergenerational conflict than in the non-intergenerational conflict; such discrepancy between conflict types was relatively small in older workers. Compared with younger workers, older workers fixated significantly less on the coworker during the intergenerational conflict scenes. A significant indirect effect of age group through visual fixation on the coworker was observed for positive emotions and avoiding. Results revealed that older workers may regulate their emotional reactions and conflict strategies to workplace conflicts by reducing their attention to negative stimuli.

## Introduction

Intergenerational and non-intergenerational workplace conflicts refer to work situations in which employees from dissimilar and similar age/generational groups, respectively, have disputes and disagreements about how work tasks are accomplished (Dencker et al., 2007). Research evidence reveals that emotional and behavioral responses to work and non-work tensions vary with the ages of the individuals or opposing party (Birditt et al., 2005; Fingerman et al., 2008; Davis et al., 2009; Yeung et al., 2020). These findings suggest that the well-documented age differences in conflict responses further vary between intergenerational and non-intergenerational conflicts in which the age group of the conflicting parties varies. Laboratory studies on visual attention have indicated that relative to younger adults, older adults prefer to look at positive stimuli and turn away from negative stimuli (Noh et al., 2011; Isaacowitz and Choi, 2012). These age-related attentional patterns have been linked to emotional outcomes; however, whether the age-related differences in attentional patterns may have consequences in real-life contexts, such as work context, remain unclear.

With a growing population of aging workers in the labor force, this study aims to extend previous research to the work context in which satisfactory conflict management is influential to team performance and the overall productivity of an organization. On the basis of the process model of emotion regulation (Gross, 2015) and the framework of linking positive looking to mood regulation by Isaacowitz (2012), which posits that an individual’s attention precedes his/her emotional and behavioral responses to an event, this study further investigates whether visual attention to workplace conflict scenes relates to age-related differences in emotional responses and conflict strategies. To address the abovementioned research questions, this work uses an experimental eye tracking paradigm to compare the visual attention of younger workers (YW) and older workers (OW) to hypothetical intergenerational and non-intergenerational workplace conflicts and assess its effects on emotional responses and conflict strategies. Advancing the current literature on workplace conflict management, which often focuses on the impacts of conflicts on employees and work-related outcomes (such as types of conflict strategies for handling conflict incidents and consequences of conflict management on team performance and satisfaction with team members; see De Dreu and Weingart (2003) for a meta-analytic review), this study’s assessment of visual attention will permit an investigation into the cognitive processes of YW and OW when observing a conflict incident involving a coworker from a similar or dissimilar age group. Understanding these cognitive processes is essential as they may play an important role in predicting subsequent interactions between two conflicting parties (DeChurch et al., 2013), such as their conflict management strategies in dealing with conflict incidents.

### Reactions to Work and Non-work Conflicts

Studies on lifespan development have revealed that compared with younger people, older people often report more positive and less negative affect even after controlling for their sociodemographic characteristics, personality, and health (Mroczek and Kolarz, 1998; Carstensen et al., 2011). When dealing with negative events or interpersonal tensions in non-work contexts, older adults often experience fewer negative emotions and are more likely to utilize passive strategies, such as avoidance and suppression, than their younger counterparts. For example, Charles et al. (2009) used data from the National Study of Daily Experiences and found that older people report fewer negative emotions than their younger counterparts when interpersonal tensions occur. Similarly, Scheibe et al. (2019) utilized a daily diary approach and demonstrated that older Chinese and German workers react to negative work events less negatively than their younger peers.

Rahim (1983, 2011) identified five types of conflict strategies to understand one’s ways of handling disputes and disagreements with other individuals: integrating, compromising, dominating, obliging, and avoiding. In particular, integrating aims to reach an effective solution between two parties, such as open discussion and exchange of information with the other party to find a solution. Compromising is utilized when the two parties aim to identify a mutually acceptable agreement. Integrating and compromising are often grouped together and labeled as constructive strategies because of their common goal of reaching a solution that is acceptable to both parties (e.g., Davis et al., 2004; Yeung et al., 2020). Dominating involves the use of forcing behaviors, such as confrontations, to satisfy one’s own needs. Obliging refers to the attempts that emphasize the commonalities between two parties and de-emphasize one’s own need to satisfy the other party’s needs, such as accommodating and yielding. Avoiding is used when an individual neglects his/her needs and those of others, such as withdrawal and sidestepping the situation.

In an examination of age-related differences in behavioral responses to conflicts with colleagues, Davis et al. (2009) found that OW are more likely than their younger counterparts to adopt passive-avoidant strategies (such as delay responses and adapting). They also found that workers aged 55 years and above are less likely than their younger counterparts to engage in dominating. Similarly, in a recent daily diary study, older employees were found to utilize more avoiding and obliging than their younger peers when handling workplace conflicts (Yeung et al., 2020).

The findings from work and non-work contexts indicate that compared with younger individuals, older individuals tend to report fewer negative emotions and utilize more avoiding and obliging and fewer dominating when handling tensions with others.

### Reactions to Intergenerational Conflicts

Social identity theory (SIT; Tajfel and Turner, 1985) posits that people tend to classify themselves and others on the basis of their perceived similarities and differences (such as age group and gender). These categorizations influence their attitudinal and behavioral tendencies when encountering conflicts. Individuals tend to assign positive attributes to in-group members (e.g., coworkers of similar ages) and negative attributes to out-group members (e.g., coworkers of dissimilar ages; Bizman and Yinon, 2004). Dencker et al. (2007) applied this theory to intergenerational conflicts and proposed that when an opposing party is from a similar age group, individuals tend to identify him/her as an in-group member and are likely to cooperate. When the opposing party is from a dissimilar age group, individuals are likely to perceive him/her as an out-group member and exhibit assertive and destructive responses. Ho and Yeung (2019) manipulated working adults’ group identity in an experimental study and revealed that the participants in the age group categorization condition had a greater tendency to use dominating and a lower tendency to use obliging than the participants in other conditions without age group categorization. These findings suggest that emotional responses and conflict strategies vary by types of conflict, with more negative and destructive responses to conflicts involving parties of dissimilar ages (hereafter labeled as intergenerational conflicts) than conflicts involving parties of similar ages (hereafter labeled as non-intergenerational conflicts).

Despite social categorization, the characteristics of the opposing party also affect one’s responses to a conflict incident. For instance, in a community sample, younger and older American adults displayed more negative responses (e.g., more upset and blame) and confrontational behaviors when encountering tension with younger social partners than with older social partners (Fingerman et al., 2008). Another study using a daily diary design revealed that Chinese employees, regardless of their age, reported more positive emotions and passive strategies when dealing with an older opposing party than with a younger opposing party (Yeung et al., 2020). The findings of the two studies offer an alternate view to the proposition of SIT, that is, responses to intergenerational conflicts are not always negative but would vary depending on the age group of the parties involved in these events.

The current study therefore aims to advance the current literature on lifespan development, SIT, and conflict management by testing whether emotional responses and conflict strategies will be moderated by conflict type (intergenerational vs. non-intergenerational), the age group of workers (young vs. old), and their interaction. On the one hand, inferring from the proposition of SIT, individuals will report more negative and fewer positive emotions and use more dominating when observing a hypothetical intergenerational conflict vignette than when viewing a non-intergenerational conflict vignette. Hypothetical vignettes have been widely used in previous research on conflict with coworkers, peers, or romantic partners (e.g., Zhang et al., 2005; Keener et al., 2012; Ho and Yeung, 2019) because of their higher internal validity (i.e., the conflict situations were exactly the same across participants in previous research; Keener et al., 2012). Accordingly, this study uses hypothetical conflict vignettes to assess the emotional responses and conflict strategies of YW and OW. On the other hand, although younger adults generally show a higher level of negative responses than older adults, Fingerman et al. (2008) and Yeung et al. (2020) found that individuals of all ages react more negatively toward younger social partners than toward older social partners. These findings suggest that when dealing with intergenerational and non-intergenerational conflicts, the effect of in- and out-group categorizations may not be the same for YW and OW. Specifically, YW would display more adverse reactions to a young conflict partner than to an older conflict partner (non-intergenerational and intergenerational conflict, respectively), whereas the pattern would be reversed in OW. Accordingly, Hypothesis 1 predicts the following:

H1a: The effect of age group on emotional responses will be moderated by conflict type. In particular, OW will report higher levels of negative emotions and lower levels of positive emotions in intergenerational conflicts than in non-intergenerational conflicts. By contrast, YW will report higher levels of negative emotions and lower levels of positive emotions in non-intergenerational conflicts than in intergenerational conflicts.

H1b: The effect of age group on conflict strategies will be moderated by conflict type. Specifically, OW will use fewer avoiding and obliging and more dominating in intergenerational conflicts than in non-intergenerational conflicts. By contrast, YW will utilize more avoiding and obliging and less dominating in intergenerational conflicts than in non-intergenerational conflicts. The use of integrating and compromising (constructive strategies) will be comparable between the two age groups in both conflict types.

### Visual Attention to Affective Stimuli

According to the process model of emotion regulation by Gross (2015), emotion-generative processes follow a situation–attention–appraisal–response sequence, which suggests that attentional deployment can predict an individual’s emotional responses and conflict strategies to an event. Negative responses, such as angry facial expressions and aggressive body movements, are commonly observed in both parties of a conflict event. These cues play critical roles in the evaluation of conflict situations and subsequently influence the affective and behavioral reactions of the parties involved. For example, in a hypothetical conflict with an instructor, Zigarovich and Myers (2011) found that undergraduate students utilized competing when they perceived their instructor to be verbally aggressive, whereas they tended to use collaborating and compromising when they perceived the instructor to be caring and verbally unaggressive. Slessor et al. (2010) also demonstrated that gaze direction could affect the judgment of angry and joyful faces. These findings suggest that how a person attends to contextual cues might influence his/her subsequent reactions to conflict.

Previous studies on visual attention have indicated that compared with their younger counterparts, older adults attend more to positive stimuli and less to negative stimuli (Isaacowitz et al., 2006; Isaacowitz and Choi, 2012; see Reed et al., 2014 for a meta-analytic review). For example, a recent study revealed that older German adults exhibited fixation patterns away from negative images (Wirth et al., 2017). When viewing slides of angry and sad facial expressions, younger adults spent a longer time looking at the eyes of an actor compared with their older counterparts (Murphy and Isaacowitz, 2010). In accordance with socioemotional selectivity theory (SST; Carstensen, 2006), the age-related shift toward positive information and away from negative information is called “age-related positivity effect.” Such an effect in visual attention has been conceptualized to be driven by one’s perception of limited future time, thereby motivating older adults to focus more on emotion-related goals than on knowledge-related goals.

The age-related differences in visual attention have been linked to emotional outcomes (see Isaacowitz, 2012 for a review); however, most of existing studies have utilized only synthetic emotional faces (e.g., Isaacowitz et al., 2009) or negatively valenced pictures from the International Affective Picture System (e.g., Noh et al., 2011) as test stimuli. Hence, no study has ascertained whether age-related differences in attention are also prevalent in real-life contexts in which emotional situations are encountered, such as workplace conflicts. The unsatisfactory handling of workplace conflicts harms the relationships among coworkers and impedes the overall productivity of an organization (De Dreu, 2011). Therefore, this study intends to extend the investigation to the work context and examine whether the age-related differences in visual attention are moderated by the type of workplace conflict involving parties from similar and dissimilar age groups. This study also investigates whether the age-related differences in visual attention could explain the variations in emotional responses and conflict strategies between YW and OW.

Research on workplace conflict has shown that OW tend to utilize avoidance to a greater extent than YW (Davis et al., 2009; Yeung et al., 2015a, 2020). Qualitative studies have also revealed that OW tend to avoid negative interactions with younger coworkers (Urick et al., 2017). These behavioral differences between age groups imply that relative to YW, OW will be more likely to look less at the opposing party (hereafter labeled as the “coworker”) in an intergenerational conflict than in a non-intergenerational conflict. Building on the framework of SIT, van Bavel and Cunningham (2012) demonstrated that a viewer’s sustained attention to in-group faces is longer than that to out-group faces. Accordingly, one can expect that when viewing an intergenerational conflict involving a main actor and a coworker, the viewer will fixate less on the coworker of a dissimilar age (an out-group member) than on the main actor of a similar age (an in-group member). Such an effect is less prevalent in a non-intergenerational conflict involving the two parties of similar age.

Integrating the literature on aging and visual attention, SIT, and conflict management together, this work derives H2:

H2: The effect of age group on fixation time on the main actor and coworker will be moderated by conflict types. When viewing an intergenerational conflict, OW will fixate less on the coworker than YW. By contrast, such age difference would be less prevalent when viewing a non-intergenerational conflict. Fixation time on the main actor in intergenerational and non-intergenerational conflicts will be similar between the two age groups.

Drawing from prior findings on the age-related differences reviewed above, an individual’s attention to affective stimuli may partly determine his/her subsequent emotional or behavioral reactions to conflict (see Isaacowitz, 2012 for a review). This study therefore takes a step further and examines whether the age effect on visual fixation can explain the age-related variations in emotional responses and conflict strategies (as hypothesized in H1).

H3: Age is associated indirectly with emotional responses and conflict strategies through fixation time on two parties when observing a workplace conflict. In particular, compared with YW, OW will fixate less on the younger coworker in an intergenerational conflict. Such fixation will subsequently contribute to the experience of more positive but fewer negative emotions and to the utilization of more avoiding and obliging and fewer dominating.

### The Present Study

This study has two key objectives: to investigate whether the levels of emotional responses and conflict strategies will be moderated by the age group of workers, conflict type, and their interaction (H1-H2) using an experimental paradigm; and to test whether the attentional patterns can explain the age-related variations in emotional responses and conflict strategies (H3). The findings of this study will contribute to the literature on aging and organizational psychology by examining whether the age-related differences in visual attention, as revealed in the existing laboratory studies, may also have other real-world consequences. The three hypotheses were tested by conducting an eye tracking study among younger and older working adults. In the eye tracking study, two hypothetical workplace task conflict videos were shown to each participant. The use of hypothetical conflict vignettes helps to reduce the ambiguity of the perceived age group of the coworker in the intergenerational conflict, as well as to control for the nature of the conflict incident (Keener et al., 2012), thus allowing an objective comparison between the two groups of working adults. Even though the participants did not directly experience a conflict in this study, previous eye tracking research has demonstrated similarities between gaze in the real world and gaze in watching a video in the laboratory (Foulsham et al., 2011). Therefore, the current study design enables us to understand how YW and OW behave in the real world through the ways they fixate on the main actor and coworker when viewing intergenerational and non-intergenerational conflict vignettes.

In Hong Kong, individuals aged over 40 years are eligible to apply for training allowance through the Employment Program for the Elderly and Middle-Aged. Therefore, the OW in this study refer to those aged over 40 years while the YW are those aged below 35 years. A point worth noting is that the OW in this study are younger than the typical older people (whose age is 65 years and above) in the research on aging and lifespan development.

## Materials and Methods

### Participants

Younger workers aged below 35 years and OW aged over 40 years were invited to participate in this study. A total of 94 Chinese employees working full-time or part-time in Hong Kong successfully completed the eye tracking experiment, with 48 YW (*M* = 28.65 years, *SD* = 3.58, range = 20–34; 50% women) and 46 OW (*M* = 53.26 years, *SD* = 7.11, range = 40–68; 56.5% women). This sample size has sufficient power to detect the small effect of age group on visual attention and responses to workplace conflicts (Repeated Measures ANOVA: within-between interaction: 2 age groups, 2 measurements, effect size = 0.20, power = 95%; α = 0.05; G^∗^Power program; Faul et al., 2007).

In the current sample, the YW had a higher education level than the OW [χ*^2^*(1,94) = 20.80, *p* < 0.001]. In particular, 93.8% of the YW had completed a bachelor’s or master’s degree, whereas the majority of the OW (52.2%) completed secondary school education. These age-related differences in education level are comparable to those observed in the Hong Kong population (Census and Statistics Department, 2017). The two age groups were similar in their occupations [1 = professional and managerial employees (51.1%); 2 = clerical staff and others (48.9%); χ*^2^*(1,94) = 0.38, *p* = 0.54]. The participants were also asked to report their job position (1 = without a supervisory role; 2 = with a supervisory role). Half of the OW reported having a supervisory role, whereas only 25% of the YW had such responsibility [χ*^2^* (1,94) = 6.28, *p* = 0.012].

### Video Stimuli

Two hypothetical videos were used to depict intergenerational and non-intergenerational conflicts between two same-gender workers in a work setting. Two video vignettes were created by the research team to portray task conflict incidents (Jehn, 1994; de Wit et al., 2012) on the basis of the literature on workplace conflicts (Hartwick and Barki, 2002; Zhang et al., 2005) and the field experiences of six local employees working in diverse industries (e.g., banking, retail, and education). Self-developed video vignettes can standardize test stimuli being presented to participants, minimize the potential familiarity effects associated with well-known actors/actresses, and facilitate mundane realism (Blascovich et al., 2002). Experienced actors/actresses were recruited through a footage company. They wore plain outfits to minimize participants’ distractions while watching the videos. Each actor/actress only appeared in one video.

The intergenerational conflict video involved a young actor/actress aged below 30 years and an older actor/actress aged around 50 years. The non-intergenerational conflict video involved actors of the same age group. In each video, the main actor matched the age and gender group of the participant to enhance the participants’ perceived relevance of the conflict situation. Accordingly, four versions (2 genders × 2 age groups) were produced for each conflict vignette with the same conflict content and script: Versions 1, 2, 3, and 4 in which the main actors are a young female, an older female, a young male, and an older male, respectively. In each version, the main actor and coworker are of the same gender. Before the actual study, **a pilot study was conducted among 26 working adults** (14 YW and 12 OW) to ensure the internal validity of the four versions of each conflict video. These participants were asked to evaluate whether the four versions of each video were similar in the intensity of negativity using a rating scale ranging from –3 (very negative) to +3 (very positive). Neither the ratings of the four versions of each conflict video [Intergenerational conflict video: *F*(3,21) = 0.86, *p* = 0.479; Non-intergenerational conflict video: *F*(3,21) = 0.91, *p* = 0.452] nor the three-way interaction effect among version, video, and age group [*F*(3,21) = 0.29, *p* = 0.835] were statistically different. The majority of the participants indicated that they had experienced similar conflict events in their own jobs. The results of this pilot study demonstrate that the four versions of the two conflict videos are comparable in their levels of negative valence and that the two videos can portray real-life conflicts in the workplace. The Supplementary Materials provides a snapshot of the four versions of the intergenerational conflict video and the URL links to the sample videos.

The main actor and coworker in each conflict were portrayed as peers (in the same job rank) regardless of their age to minimize the potential confounding effect of power. In the intergenerational conflict video, the main actor and coworker from different age groups dispute over how to handle a new sales order. In the non-intergenerational conflict videos, the two parties of the same age group show disagreement over the design of a poster for promoting a new product. Despite the differences in arguments, the two video vignettes share a common theme, that is, organizational productivity, thus affecting both parties in the conflict. These videos first open with the main actor, and then the coworker enters the scene and questions the way the main actor handles his/her work task. The main actor expresses his/her views but fails to reach an agreement with the coworker. Relative to the main actor, the coworker is portrayed as more negative and unfriendly in the video. Each video clip was less than 60 s long.

### Equipment

An Applied Science Laboratories (Bedford, MA, United States) Model D6 eye tracker desktop/remote optics was used to record the participants’ eye movements and fixation patterns at a rate of 120 Hz. Gaze-Tracker software (Eye Response Technologies, Charlottesville, VA, United States) was used to present the stimuli on a 19-inch monitor and to facilitate the recording, storage, and analysis of the data.

### Procedure

Research ethics approval was obtained from the Human Subjects Ethics Sub-Committee of the affiliated university of the first author. All participants completed an online baseline questionnaire before taking part in the laboratory session. Written consent was obtained from each participant before the eye tracking study. The visual acuity of the participants was tested by conducting a Snellen visual acuity test and a near vision test (Lam et al., 2008). A nine-point calibration was also performed before showing the videos.

The two videos were presented in random order. The participants were asked to imagine themselves as the first actor appearing in these videos (i.e., the main actor). After watching each video, the participants were asked to answer an online questionnaire that recorded their emotional responses to the conflict depicted in the video and their conflict strategies to manage this incident if they were the main actor in the vignette. The entire experiment lasted for approximately 30 min. All participants received supermarket vouchers worth HKD150 (USD19) as compensation for their participation.

### Eye Tracking Measures

#### Percentage of Fixation on the Main Actor and Coworker During the Conflict Scene

For each video, two areas of interest (AOIs) were set while two actors were having a disagreement over their work task (i.e., the conflict scene). The first AOI was on the face of the main actor, and the second AOI was on the face of the coworker. The percentage of fixation on an AOI was computed by dividing the total fixation time on the actor by the total showtime of the conflict scene.

### Post-video Questionnaire Measures

#### Emotional Responses

After watching each video, the participants were asked to indicate their levels of positive and negative emotions in response to the conflict incident. With reference to prior studies on workplace affect and conflict management (Yeung and Fung, 2012; Scheibe et al., 2019), six items were used to measure positive emotions (happy, calm, enthusiastic, relaxed, joyful, and excited), and seven items were used to measure negative emotions (angry, irritated, worried, sad, frustrated, dissatisfied, and anxious). These 13 items were rated on a 7-point scale, ranging from 1 = none to 7 = extremely, with higher scores representing more positive or negative emotions. The mean scores of positive and negative emotions were computed separately for the intergenerational and non-intergenerational conflicts. The Cronbach’s alphas of positive emotions in the intergenerational and non-intergenerational conflicts were 0.80 and 0.84, respectively; whereas the alphas of negative emotions in these two conflicts were 0.90 and 0.91, respectively.

#### Conflict Strategies

The participants were also asked to indicate their way of handling the incident if they were the main actor in the conflict video. Five items were selected from the Rahim Organizational Conflict Inventory-II (ROCI-II; Rahim, 1983; Yeung et al., 2015a) to assess the participants’ way of handling the conflict depicted in the video. The original ROCI-II measures five types of conflict responses, namely, integrating (“I try to work with the coworker to find solution to a problem that satisfies our expectations”), compromising (“I usually propose a middle ground for breaking deadlocks”), dominating (“I am generally firm in pursuing my side of the issue”), avoiding (“I try to keep my disagreement with the coworker to myself in order to avoid hard feelings”), and obliging (“I usually accommodate the wishes of the coworker”). However, measuring all 28 items for each video would make the entire experiment excessively long. Hence, the item that obtained the highest factor loading was selected from each of the five conflict types on the basis of previous studies on Chinese working adults (Yeung et al., 2015a). Each item was rated on a 7-point scale, ranging from 1 = very unlikely to 7 = very likely, with higher scores indicating greater tendency to utilize the conflict strategy. With reference to previous research (Davis et al., 2009; Yeung et al., 2020), the scores of integrating and compromising were averaged into constructive strategies. For the other three types of conflict strategies, they were measured only by one item. The Cronbach’s alphas of the constructive strategies in the intergenerational and non-intergenerational conflicts were 0.84 and 0.72, respectively.

### Demographic Variables and Covariates

The age, gender, education, occupation, and job position of the participants were recorded in the baseline questionnaire.

### Analytical Plan

To test H1 and H2, Repeated Measures ANCOVAs were conducted separately on emotional responses, conflict strategies, and fixation patterns, with the age group of the participants as a between-subjects variable and the conflict type and/or actors as within-subjects variables. Mediation analyses were conducted to test H3, that is, whether age is associated indirectly with emotional responses and conflict strategies through visual attention. In all the analyses, gender and job position were controlled as covariates because research has shown that workers of different genders and job positions utilize conflict strategies differently (Davis et al., 2009; Yeung et al., 2020). The results remained largely the same even when these covariates were not controlled in the analyses.

## Results

Table 1 summarizes the means and standard errors (*SE*) of emotional responses and conflict strategies in the intergenerational and non-intergenerational conflicts for each age group.

**TABLE 1:** Descriptive statistics of emotional responses, conflict strategies, and visual fixation.

	Intergenerational Conflict		Non-intergenerational Conflict	
	Mean (*SE*)		Mean (*SE*)	
	Younger Workers	Older Workers	Younger Workers	Older Workers
**Emotional responses**				
Positive emotions	1.94 (0.13)	2.39 (0.13)^a^	2.06 (0.14)	2.65 (0.14)^b^
Negative emotions	4.40 (0.19)	4.03 (0.20)	4.06 (0.20)	3.33 (0.20)^b^
**Conflict strategies**				
Constructive strategies	4.90 (0.18)	5.38 (0.158)	5.02 (0.15)	5.44 (0.15)
Obliging	3.32 (0.22)	3.58 (0.22)	4.03 (0.19)	4.53 (0.20)
Dominating	4.99 (0.21)	4.23 (0.21)^a^	3.59 (0.20)	3.48 (0.21)
Avoiding	3.25 (0.23)	3.42 (0.24)	3.90 (0.24)	3.15 (0.24)^b^
**Percentage of visual fixation**				
Fixation on the main actor	0.22 (0.02)	0.22 (0.02)	0.35 (0.02)	0.28 (0.02)^b^
Fixation on the coworker	0.39 (0.02)	0.28 (0.02)^a^	0.33 (0.02)	0.29 (0.02)

*Adjusted mean after controlling for gender and job position. ^a^Represents mean scores of younger and older workers in the intergenerational conflict are statistically different from each other (p < 0.05). ^b^Represents mean scores of younger and older workers in the non-intergenerational conflict are statistically different from each other (p < 0.05).*

### Effects of Conflict Type and Age Group on Emotional Responses

To test H1a, a 2 [type (intergenerational vs. non-intergenerational)] × 2 [emotion (positive vs. negative) × 2 [age group (young vs. older)] Repeated Measures ANCOVA was conducted with conflict type and emotion as within-subjects factors and age group as a between-subjects factor.

The three-way interaction effect among conflict type, emotion, and age group was not significant [*F*(1,90) = 3.41, *p* = 0.068, $\eta_{\text{p}}^{2}$ = 0.036], but the two-way interaction between emotion and age group was significant [*F*(1,90) = 12.03, *p* = 0.001, $\eta_{\text{p}}^{2}$ = 0.118]. To understand the significant two-way interaction effect, Repeated Measures ANCOVAs were conducted on positive and negative emotions separately. In terms of positive emotions, the main effect of age group was significant [*F*(1,90) = 9.163, *p* = 0.003, $\eta_{\text{p}}^{2}$ = 0.092]. Specifically, the OW reported more positive emotions than the YW in the intergenerational [*M_*intergen_ow*_* = 2.39, *SE* = 0.13; and *M_*intergen_yw*_* = 1.94, *SE* = 0.13; *t*(92) = 2.54, *p* = 0.013] and non-intergenerational conflicts [*M_*non–intergen_o*__*w*_* = 2.65, *SE* = 0.14; and *M_*non–intergen_yw*_* = 2.06, *SE* = 0.14; *t*(92) = 3.03, *p* = 0.003].

**FIGURE 1:**
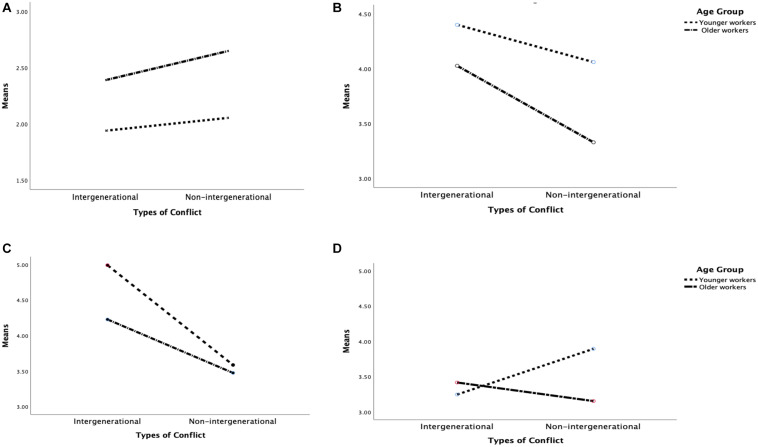
Interaction effects between conflict type and age group on emotions and conflict strategies. **(A)** Positive Emotions; **(B)** Negative Emotions; **(C)** Dominating; and **(D)** Avoiding.

In terms of negative emotions, the main effect of age group was significant [*F*(1,90) = 4.551, *p* = 0.034, $\eta_{\text{p}}^{2}$ = 0.048]. Specifically, both YW and OW reported similar levels of negative emotions after observing the intergenerational conflict, but OW reported significantly fewer negative emotions than YW after viewing the non-intergenerational conflict [*M_*non–intergen_ow*_* = 3.33, *SE* = 0.20, and *M_*non–intergen_yw*_* = 4.06, *SE* = 0.20; *t*(92) = 2.62, *p* = 0.011]. Figures 1A,B illustrate the age differences in emotional responses.

In sum, the OW generally reported better affective well-being (with more positive emotions and fewer negative emotions) than the YW after observing workplace conflicts involving coworkers from similar and dissimilar age groups. The two age groups reported similar levels of negative emotions in the intergenerational conflict, but the OW reported significantly fewer negative emotions than the YW in the non-intergenerational conflict. As no significant three-way interaction effect was shown, H1a was not supported.

### Effects of Conflict Type and Age Group on Conflict Strategies

Similar to H1a, a 2 [type (intergenerational vs. non-intergenerational)] × 4 [strategy (constructive vs. obliging vs. avoiding vs. dominating) × 2 [age group (young vs. older)] Repeated Measures ANCOVA was conducted to test H1b. The three-way interaction effect among conflict type, strategy, and age group was significant [*F*(3,88) = 4.08, *p* = 0.009, $\eta_{\text{p}}^{2}$ = 0.122]. To understand this interaction effect, Repeated Measures ANCOVAs were conducted on each of the four conflict strategies separately. The results showed that the effects of age group on dominating [*F*(1,90) = 4.00, *p* = 0.049, $\eta_{\text{p}}^{2}$ = 0.043] and avoiding [*F*(1,90) = 7.96, *p* = 0.006, $\eta_{\text{p}}^{2}$ = 0.081] were significantly moderated by conflict type. However, such interaction effects were not observed on the constructive strategies and obliging. Consistent with the prediction, the OW utilized more dominating in the intergenerational conflict than in the non-intergenerational conflict [*t*(45) = 3.06, *p* = 0.004]; however, their use of avoiding was similar across the two conflicts [*t*(45) = 1.32, *p* = 0.194]. Contrary to the prediction, the YW used more dominating [*t*(47) = 6.43, *p* < 0.001] and fewer avoiding [*t*(47) = –2.60, *p* = 0.012] in the intergenerational conflict than in the non-intergenerational conflict. Figures 1C,D illustrate the interaction effects on these two conflict strategies.

A significant main effect of age group was shown in the use of dominating in the intergenerational conflict and the use of avoiding in the non-intergenerational conflict. Compared with the OW, the YW used more dominating in the intergenerational conflict [*t*(92) = 2.64, *p* = 0.010], and utilized more avoiding in the non-intergenerational conflict [*t*(92) = 2.09, *p* = 0.040].

In sum, both age groups used more dominating in the intergenerational conflict than in the non-intergenerational conflict, but the intensity of usage of the YW was significantly higher than that in the OW. When dealing with the non-intergenerational conflict, the YW tended to use avoiding less than the OW did. The use of constructive strategies and obliging did not differ by age group or conflict type. Therefore, H1b was partly supported.

### Effects of Conflict Type, Actor, and Age Group on Visual Fixation

H2 proposes an interaction effect among conflict type, actor, and age group on the percentage of fixation. This hypothesis was tested by conducting a 2 [type (intergenerational vs. non-intergenerational)] × 2 [actor (main actor vs. coworker)] × 2 [age group (young vs. older)] Repeated Measures ANCOVA on the percentage of fixation on the two actors during the conflict scene.

**FIGURE 2:**
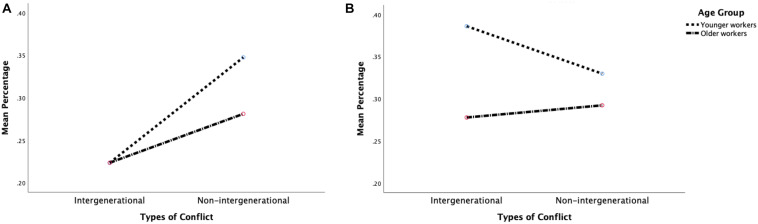
Interaction effects between conflict type and age group on visual attention on the main actor **(A)** and coworker **(B)**.

The three-way interaction effect among conflict type, actor, and age group was significant [*F*(1,90) = 8.53, *p* = 0.004, $\eta_{\text{p}}^{2}$ = 0.087], thus indicating that the fixation patterns of the YW and OW toward the two actors varied by conflict type. In particular, when viewing the intergenerational conflict, the OW fixated significantly less on the coworker than the YW [*M_*o*__*w*_* = 0.28, *SE* = 0.02; and *M_*y*__*w*_* = 0.39, *SE* = 0.02; *t*(92) = 3.31, *p* = 0.001], but such an age difference was not shown when viewing the main actor. By contrast, in the non-intergenerational conflict, age differences were only observed when viewing the main actor, with the YW showing longer fixation time than the OW [*M_*y*__*w*_* = 0.35, *SE* = 0.02; and *M_*o*__*w*_* = 0.28, *SE* = 0.02; *t*(92) = 2.11, *p* = 0.037]. Figures 2A,B illustrate these interaction effects on visual fixation.

A significant main effect of age group [*F*(1,90) = 5.34, *p* = 0.023, $\eta_{\text{p}}^{2}$ = 0.056] was also observed, with the OW on average showing less fixation on the two actors than the YW.

In sum, during conflict situations, the OW on average paid less attention to the main actor and coworker than the YW did. This age effect was moderated further by conflict type, with the OW showing less fixation on the coworker than the YW did in the intergenerational conflict but not in the non-intergenerational conflict. Therefore, H2 was partly supported.

### Indirect Effect of Age on Emotional Responses and Conflict Strategies Through Fixation

The aforementioned results revealed that the age differences in emotional responses, conflict strategies, and fixation were observed mainly in the intergenerational conflict. Therefore, the indirect effects of age on emotional responses and conflict strategies were tested through the fixation scores in the intergenerational conflict by using PROCESS Model 4 (Hayes, 2019). Age group (1 = YW; 2 = OW) was entered as the independent variable in the regression model, visual fixations on the main actor and coworker were entered as M variables, and the positive and negative emotions, dominating, and avoiding were entered separately as dependent variables.

In the intergenerational conflict, age had significant indirect effects on positive emotions [*B* = 0.21, *SE* = 0.10 (0.05,0.43)] and avoiding [*B* = 0.27, *SE* = 0.15 (0.03,0.61)] through fixation on the coworker, thereby suggesting that the OW reported more positive emotions and utilized more avoiding to manage an intergenerational conflict as they fixated less on the coworker in comparison with their younger counterparts. However, the indirect effects of age on negative emotions or dominating through fixation on the two parties were not significant. The results of the indirect effects of age group on the four outcome variables are summarized in Table 2. These results partly support H3. When viewing an intergenerational conflict, the OW fixated less on the coworker of a dissimilar age, hence resulting in their higher levels of positive emotions and avoiding in comparison with the YW.

**TABLE 2:** Results of the indirect effect of age group on emotional responses and conflict strategies to intergenerational conflict through visual fixation.

Predictors	Positive Emotions		Negative Emotions	
	*B*	*SE*	*B*	*SE*
**DV: Fixation on the main actor**				
Constant	0.159*	0.061	0.159*	0.061
Age group	0.000	0.025	0.000	0.025
Gender	0.013	0.026	0.013	0.026
Job position	0.030	0.026	0.030	0.026
R^2^	0.021		0.021	
**DV: Fixation on the coworker**				
Constant	0.533***	0.079	0.533***	0.079
Age group	–0.108**	0.033	–0.108**	0.033
Gender	0.030	0.034	0.030	0.034
Job position	–0.047	0.034	–0.047	0.034
R^2^	0.136**		0.136**	
**DV: Emotion**				
Constant	2.244***	0.502	4.551***	0.805
Age group	0.242	0.183	–0.322	0.294
Fixation on the main actor	1.057	0.788	1.185	1.263
Fixation on the coworker	–1.916**	0.605	–0.477	0.970
Gender	–0.166	0.177	–0.468	0.284
Job position	0.142	0.183	0.295	0.293
R^2^	0.188**		0.072	
**Total, direct, and indirect effects of X on Y**	*B (Boot SE)*	*LL-UL95%CI*	*B (Boot SE)*	*LL-UL95%CI*
Total effect	0.450 (0.179)	0.095 –0.804	–0.374 (0.274)	–0.919 –0.171
Direct effect	0.242 (0.183)	–0.122 –0.607	–0.322 (0.294)	–0.906 –0.262
Indirect effect through fixation on the main actor	0.000 (0.036)	–0.078 –0.076	0.000 (0.045)	–0.104 –0.089
Indirect effect through fixation on the coworker	0.207 (0.098)	0.050 –0.434	–0.052 (0.113)	–0.299 –0.158

Age group was coded as 1 = younger workers (20–34 years) and 2 = older workers (40 years and above). Gender and job position were controlled as covariates. *p < 0.05; **p < 0.01; ***p < 0.001.				

** Predictors**	**Dominating**		**Avoiding**	
	***B***	***SE***	***B***	***SE***

**DV: Fixation on the main actor**				
Constant	0.159*	0.061	0.159*	0.061
Age group	0.000	0.025	0.000	0.025
Gender	0.013	0.026	0.013	0.026
Job position	0.030	0.026	0.030	0.026
R^2^	0.021		0.021	
**DV: Fixation on the coworker**				
Constant	0.533***	0.079	0.533***	0.079
Age group	–0.108**	0.033	–0.108**	0.033
Gender	0.030	0.034	0.030	0.034
Job position	–0.047	0.034	–0.047	0.034
R^2^	0.136**		0.136**	
**DV: Conflict strategy**				
Constant	5.965***	0.883	3.826***	0.923
Age group	–0.683	0.322	–0.097	0.337
Fixation on the main actor	–0.943	1.386	–1.702	1.449
Fixation on the coworker	–0.082	1.064	–2.479	1.113
Gender	–0.008	0.311	0.388	0.326
Job position	–0.238	0.321	0.160	0.336
*R*^2^	0.088		0.125*	
**Total, direct, and indirect effects of X on Y**	*B (Boot SE)*	*LL-UL95%CI*	*B (Boot SE)*	*LL-UL95%CI*
Total effect	–0.763 (0.299)	–1.347 – –0.169	0.171 (0.329)	–0.483 –0.824
Direct effect	–0.683 (0.322)	–1.323 – –0.042	-0.098 (0.337)	–0.768 –0.573
Indirect effect through fixation on the main actor	0.000 (0.031)	–0.079 –0.056	0.000 (0.053)	–0.115 –0.117
Indirect effect through fixation on the coworker	–0.054 (0.081)	–0.221 –0.100	0.268 (0.150)	0.032 –0.612

Age group was coded as 1 = younger workers (20–34 years) and 2 = older workers (40 years and above). Gender and job position were controlled as covariates. *p < 0.05; **p < 0.01; ***p < 0.001.

## Discussion

By measuring the visual attention of YW and OW to intergeneration and non-intergenerational conflict scenes, emotional responses, and conflict strategies, this study considered the role of conflict type and how visual attention might underlie age-related differences in predicting emotional responses and conflict strategies when observing task conflicts in a work environment. Specifically, the levels of negative emotions of the OW were lower than those of the YW when the coworker was from a similar age group (i.e., non-intergenerational conflict), whereas such difference was not shown in the intergenerational conflict. Compared with the OW, the YW utilized more dominating in the intergenerational conflict and more avoiding in the non-intergenerational conflict. Advancing previous studies which were mainly conducted in non-work laboratory contexts (Noh et al., 2011; Isaacowitz and Choi, 2012), the current work revealed that visual attention to negative real-life work stimuli varies by age group. Specifically, the OW fixated significantly less on the scenes involving arguments and disagreements between the main actor and coworker in comparison with the YW; such age-related differences were moderated further by conflict type. Supporting our prediction, visual fixation on the coworker in the intergenerational conflict was shown to relate to age-related increases in positive emotions and avoiding.

### Emotional Responses to Intergenerational Conflict

This study advances our understanding of the affective experiences of working adults by comparing their emotional reactions to conflict incidents involving coworkers from similar and dissimilar age groups. The OW on average reported more positive and fewer negative emotions than their younger peers when observing workplace conflicts. This age effect is consistent with that in past studies in non-work (Birditt et al., 2005; Charles et al., 2009; Luong and Charles, 2014) and work contexts (Scheibe et al., 2019; Yeung et al., 2020), thereby providing additional evidence that improved affective outcomes are shown in older individuals even in the face of intergenerational and non-intergenerational conflicts.

The OW reported significantly fewer negative emotions than their younger counterparts after observing the non-intergenerational conflict, whereas both age groups reacted with more negative emotions when the coworker was from a dissimilar age group (i.e., an intergenerational conflict). These results supported the proposition of SIT (Tajfel and Turner, 1985) that social categorization is associated with more negative responses to an out-group member than to an in-group member. The stronger differences between the OWs’ emotional responses to the intergenerational conflict and the non-intergenerational conflict (see Figure 1B) imply that categorizing coworkers into in- and out-groups by age group is more prevalent in the OW than in the YW. The categorization in turn affects their emotional reactions toward coworkers from diverse age groups. Therefore, the context of workplace conflict (e.g., age of the coworker, the likelihood of social categorization) should also be considered when examining age differences in emotional responses in future research. Despite the abovementioned effect of conflict type, the overall level of negative emotions of the OW across the two conflicts was still lower than that of the YW. This result provides additional support to the previous findings on age-related advantage in affective well-being in work settings (Scheibe et al., 2016, 2019; Yeung et al., 2020).

### Conflict Management in Intergenerational Conflict

This study also advances prior works on conflict management by examining whether age differences in conflict strategies would be different in conflicts involving coworkers from similar and dissimilar age groups. Consistent with past research (Davis et al., 2009; Ho and Yeung, 2019), this study indicated that the levels of use of constructive strategies were comparable between the YW and the OW, regardless of conflict type. The results also showed that the two groups of workers preferred dominating more when dealing with the intergenerational conflict than when dealing with the non-intergenerational conflict. These findings are consistent with the proposition of SIT and the results of previous studies on working adult samples (Dencker et al., 2007; Ho and Yeung, 2019). When one perceives the coworker from a dissimilar age group as an out-group member, he/she is likely to adopt confrontational strategies to handle the conflict and protect the interests of the in-group. Such an effect of social categorization was more prevalent in the YW than in the OW, thereby implying that more escalating reactions would be observed in YW than in OW when an intergenerational conflict occurs in the workplace. Management personnel should thus be attentive to conflicts involving coworkers of different age groups and deploy appropriate measures to resolve them.

In contrast to the findings of Fingerman et al. (2008), the YW in the current sample did not show less dominating when dealing with an older coworker. The discrepancy between the two studies can be ascribed to the sample characteristics (community vs. working sample) and the relationship with the conflicting partner (close social partners vs. coworkers). It is possible that emotional closeness with the conflicting partner may influence one’s selection of conflict strategies. Future research should therefore compare individuals’ strategy usage between family- and work-related conflicts.

Inconsistent with the previous findings from work and non-work contexts (Birditt et al., 2005; Davis et al., 2009), the results of this study suggested that the OW did not always hold a higher preference for avoiding than their younger peers, at least when dealing with workplace conflicts. By contrast, the YW in the current sample showed a higher utilization of avoiding when the coworker was of similar age. On the one hand, avoiding discussion or interaction with the coworker cannot resolve the dispute at all, thereby hindering YWs’ career development in the long run. On the other hand, avoidance can help minimize YWs’ direct confrontation with the coworker, thereby contributing to a harmonic relationship with their coworkers. In the Chinese work setting, maintaining social harmony is particularly important (Ma, 2007; Yuan, 2010), especially for YW who are in the early stage of career development. A recent study found that YW’s use of passive strategies (including avoiding and obliging) is associated with fewer negative post-conflict relationship outcomes (Yeung et al., 2020), thereby suggesting a beneficial effect of utilizing avoiding in the work setting. That study also demonstrated that the usage of passive strategies is predicted by one’s perceived opportunity of future interaction with the coworker. Relative to an older coworker, YW are likely to expect more future interaction opportunities with a younger coworker; thus, avoiding is preferred to minimize direct confrontation with the younger coworker. Future research should thereby measure the perceived opportunity of future work relationship to accurately predict one’s choice of conflict strategies when handling workplace conflict.

### Visual Fixation in Intergenerational Conflict

Previous studies on lifespan development have shown that older people look at negative stimuli less frequently than their younger counterparts (Isaacowitz et al., 2006; Wirth et al., 2017). This study extends these past findings to the work context by comparing the visual attention of YW and OW to conflicts involving a coworker from similar and dissimilar age groups. The findings indicated that regardless of conflict type, the OW on average paid less attention to the conflict scenes involving arguments and disagreements between the main actor and coworker than the YW did. The aforementioned age effect was moderated further by conflict type, in which relative to the YW, the OW looked away from the younger coworker who expressed anger and disagreement in the intergenerational conflict. However, such an age effect was not shown in the non-intergenerational conflict.

Social identity theory (Tajfel and Turner, 1985) posits that social categorization influence one’s attitudes and behavioral responses. The findings of this study suggest that such categorization based on the age group of conflict partners might have occurred relatively early in the information processing of the OW and might have influenced their visual attention to the coworker in the intergenerational conflict. This age-related attentional pattern may explain the individual differences in the emotional responses and conflict strategies of individuals (as discussed further in the next section).

### Indirect Effect of Age Through Visual Fixation

On the basis of the framework of linking positive looking to mood regulation by Isaacowitz (2012), as well as the process model of emotion regulation (Gross, 2015), this study speculated that an individual’s visual attention when processing an interpersonal conflict may help explain the effect of age on his/her emotional responses. The results of the indirect effect analyses provide partial support to H3. In particular, in the intergenerational conflict, age was associated indirectly with positive emotions and avoiding through an individual’s fixation on the coworker. The OW tended to look away from the coworker who expressed anger and negativity to the main actor when processing the information in the intergenerational conflict incident. Such an attentional pattern helped them regulate their responses to the unpleasant event, consequently increasing their levels of positive emotions and the use of avoiding. These findings implied that the attentional deployment of an individual predicts his/her affective reactions and conflict strategies to workplace conflict. This work will contribute to the literature on developmental and organizational psychology by disclosing the regulatory strategy (i.e., attentional deployment) utilized by OW to maintain their affective well-being in the workplace. These psychological strengths enable OW to continue to excel at work, especially those working in service-related industries. Future training and development programs should focus on shaping the attentional patterns of working adults to promote their affective well-being.

However, no indirect effect of age was observed on dominating through visual attention to conflict partners. Other factors, such as dispositional characteristics and nature of the conflict issue (e.g., task versus relationship conflict; De Dreu and Weingart, 2003; de Wit et al., 2012), could be salient in determining one’s responses to a conflict incident. For example, individuals with higher personal negativity are likely to feel irritated or blame other people during social interactions (Furr and Funder, 1998), thereby resulting in more destructive reactions. Future studies should include these factors to obtain an enhanced picture of the underlying mechanism that contributes to age-related differences in conflict management.

### Limitations and Future Directions

Several limitations of this study must be considered when interpreting the reported findings. First, emotional responses and conflict strategies were self-reported. Thus, future studies should utilize objective measures of affective responses and conflict strategies to minimize potential bias in self-report data. Second, the actors in the videos were portrayed as coworkers. However, some participants might have perceived the older actor in the intergenerational conflict to be of a higher job position than the younger actor. Such a perception might have affected their ways of handling the incident as the findings of earlier research demonstrated the effect of the job rank of the coworker on conflict strategies (Yeung et al., 2015a). Future studies should also record the perceived relationship between the actors. Third, the data collected in this study were cross-sectional in nature, which prevented the researchers from testing the causal relationship between visual attention and emotional responses and conflict strategies. Although the link from attention to emotional responses was tested in this study, an individual’s emotional feelings may also influence his/her attention to a conflict scene. For example, Wadlinger and Isaacowitz (2011) suggested that the link between affect and attention can be bidirectional. Future studies can use experimental manipulation to uncover the causal relationship between attention and emotional responses. Fourth, because of practical concerns about the assessment duration (including calibrations, viewing of video vignettes, and post-video assessments), only one item from each of the five conflict strategy subscales of the ROCI-II was measured. Even though the ROCI-II has been adopted in previous studies and good internal consistency has been reported for each subscale (e.g., Yeung et al., 2015a, 2020; Ho and Yeung, 2020), the reliability index of avoiding, obliging, and dominating could not be computed in the present study because of the single-item measure of these strategies. Additional correlation analyses revealed that the average scores of utilizing each strategy in the intergenerational and non-intergenerational conflict vignettes (as measured in this eye tracking study) were significantly correlated with the habitual use of the respective conflict strategy (as measured in the baseline questionnaire), particularly avoiding (*r* = 0.39, *p* < 0.001), obliging (*r* = 0.21, *p* = 0.044), dominating (*r* = 0.21, *p* = 0.046). These additional data suggest that the single-item measure of each conflict strategy used in the current study could adequately capture the participants’ behavioral tendency to deal with the conflict incidents. Nevertheless, future studies should utilize the full scale of ROCI-II to comprehensively assess the employment of various conflict strategies.

Fifth, previous research has focused on assessing participants’ direct experiences in conflict situations that they have encountered personally (Davis et al., 2009; Yeung et al., 2015a). Unlike these past studies, the current study asked the participants to watch hypothetical conflict videos involving two coworkers. They were instructed to imagine themselves as the main actor in the conflict and report their own responses to the conflict incident depicted in each vignette. Such design facilitated the measure of their visual attention while observing a conflict event and minimized the potential confounds in a workplace conflict (e.g., job rank and gender of the coworker). However, their viewing patterns on the coworker and their reports of emotional responses and conflict strategies might be different from those in a typical conflict situation that they encounter personally. In particular, the data could not ascertain whether the participants reacted to the conflict by using a first-person perspective (i.e., personal involvement in a conflict situation) or third-person perspective (i.e., observing a conflict event as a third party). In a real-life conflict, individuals can only look at their conflict partner but not the main actor; for the current study, we needed to directly compare the participants’ visual attention to the coworker with that to the main actor. The same could not be achieved when using the typical first-person perspective. Whether responses would differ between the first-person and third-person perspectives awaits further investigation. With the popular use of online meetings via Zoom or Teams as the main communication means during the COVID-19 pandemic, working adults nowadays have become accustomed to seeing themselves and their coworkers simultaneously on the screen. Therefore, the findings of the present study indeed provide important insights to future studies on investigating the real-life visual processing of workplace conflicts. With the availability of portable and wearable eye trackers, future research can use such devices to replicate the current study and measure the participants’ visual processing directly in a real-time conflict situation, such as a Zoom meeting with a coworker.

Finally, the present study was conducted in a sample of Chinese working adults, whose habitual conflict management styles are different from those of their Western counterparts. For instance, DeChurch et al. (2013) found that the work teams from collectivistic cultures are less likely to adopt competition or avoidance than the teams from individualistic cultures. Hence, whether the current findings could also be generalized to non-Chinese cultural groups remains unknown and awaits further examination.

### Practical Implications

Experiencing task or relationship conflict at work is undoubtedly stressful to employees and tends to hamper their work efficiency and performance. Unsatisfactory management of intergenerational conflicts will further exacerbate the mental well-being of OW, especially given the various challenges they face at work, such as great perceived constraints and high risk of unfair treatment in personnel decision and training opportunity (Yeung et al., in press). Past research has demonstrated that chronic exposure to work-related stress, such as high job demands and frequent incidents of conflicts with coworkers, exerts detrimental impacts on the mental well-being of employees (e.g., Eskildsen et al., 2017; McCarthy et al., 2017; Giorgi et al., 2020). For example, Eskildsen et al. (2017) found that in a sample of younger and middle-aged Danish patients, changes in perceived work stress were associated with concurrent and subsequent changes in cognitive complaints over a period of 12 months. McCarthy et al. (2017) also revealed that increased job demands are correlated with high levels of depression and anxiety in a sample of older Irish health care workers. Therefore, the findings of the current study contribute to the literature on aging workers and conflict management by identifying the predictive factors of OW’s emotional reactions to intergenerational conflict, that is, how OW’s attentional pattern during a conflict incident impacts their subsequent emotional reactions and interactions with coworkers, thus influencing their mental well-being.

With age diversity becoming increasingly prevalent in organizations, employers and human resources managers should realize the differences between younger and older employees so as to reduce intergroup prejudice and the occurrence of conflict between workers from dissimilar age groups. Past research on age diversity in the workplace has mainly focused on the age differences in dispositional characteristics, such as work values and attitudes (e.g., Barnes-Farrell and Matthews, 2007; Kooij et al., 2011; Yeung et al., 2015b, 2016) and conflict management styles (e.g., Dencker et al., 2007; Urick et al., 2017; Ho and Yeung, 2020). However, little is known about the way YW and OW process a conflict incident. Contrary to the typical assumption that individuals of various age groups would show similar attention to the conflict partner when experiencing a conflict incident, this study unveils that the OW looked away from the angry face of the coworker relative to their younger counterparts. Their visual fixations during the conflict event subsequently influenced their emotional responses and selection of conflict strategies. As avoiding is associated with poor team performance (DeChurch et al., 2013), the OW should be encouraged to attend to their coworker’s emotional and behavioral reactions while interacting with one another so as to reduce their utilization of less adaptive conflict strategies such as avoiding. Training and development programs that promote intergroup contact and cooperation should also be organized to provide YW and OW more opportunities to understand and acknowledge each other’s needs and motives (Rahim, 2011) and thereby reduce intergroup prejudice and debunk stereotypical attitudes toward the other age group.

## Conclusion

This study advances the current literature on lifespan development and organizational psychology by systematically examining conflict strategies and emotional responses to conflicts involving coworkers from similar and dissimilar age groups. On average, OW experience more positive and fewer negative emotions than YW, especially after observing a non-intergenerational conflict. Relative to that in the non-intergenerational conflict, dominating is utilized in the intergenerational conflict, and such discrepancy between conflict types is more prevalent in YW than in OW. The age-related differences in emotional responses and conflict strategies can be ascribed to their visual fixation on conflict stimuli. Compared with their younger peers, the OW attended less to the coworker when observing the intergenerational conflict, thereby resulting in higher levels of positive emotions and avoiding. Consistent with earlier studies on non-work contexts, the present investigation in the work setting indicates that one way for OW to regulate their responses to interpersonal tensions is to reduce their fixation on the negative emotionally arousing stimuli. Thus, future studies on conflict management should consider conflict type and individual age when examining emotional responses and conflict strategies.

## Data Availability Statement

The raw data supporting the conclusions of this article will be made available by the authors, without undue reservation.

## Ethics Statement

The studies involving human participants were reviewed and approved by The Human Subjects Ethics Sub-Committee of City University of Hong Kong. The patients/participants provided their written informed consent to participate in this study. Written informed consent was obtained from the individual(s) for the publication of any potentially identifiable images or data included in this article.

## Author Contributions

DY contributed to the funding acquisition, conceptualization, design and implementation of the study, development of the conflict videos, statistical analysis, and writing the manuscript. DI and JY contributed to the funding acquisition, conceptualization, and reviewing the drafts of the manuscript. WL contributed to the development of the conflict videos, data collection and data cleaning, and reviewing the drafts of the manuscript. CL contributed to the data collection and reviewing the drafts of the manuscript. All authors contributed to the article and approved the submitted version.

## Conflict of Interest

The authors declare that the research was conducted in the absence of any commercial or financial relationships that could be construed as a potential conflict of interest.
