# Supplementary material for: Age Differences in Visual Attention and Responses to Intergenerational and Non-intergenerational Workplace Conflicts
Source: Front Psychol. 2021 Jun 7;12:604717. doi: 10.3389/fpsyg.2021.604717 (PMC8215446; doi:10.3389/fpsyg.2021.604717)
Supplement: Supplementary file 1 [file Presentation_1.pdf]

Supplementary Materials on Conflict Videos

The four versions of each conflict vignette are: The main actor in Version 1 is a young female; Version 2 presents an older female; Version 3 includes a young male; and Version 4 includes an older male. A snapshot for each version of the intergenerational conflict video is provided below. In the experiment, the participant was given the version in which the main actor matched the age and gender group of the participant. Each participant viewed two conflict videos. Thus, 8 videos were produced for this study (2 types of conflict video × 2 genders × 2 age groups).

A snapshot of the four versions of the intergenerational conflict video:

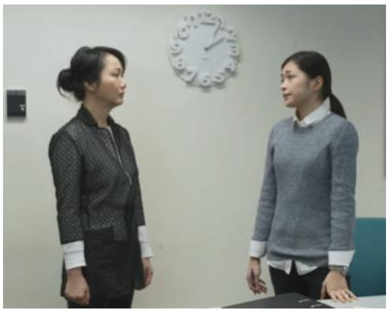

a) Version 1 was shown to younger female participants

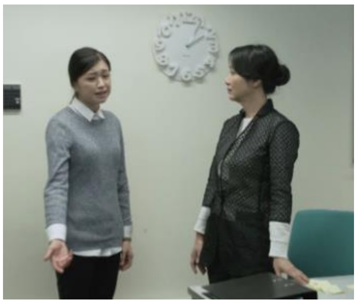

b) Version 2 was shown to older female participants

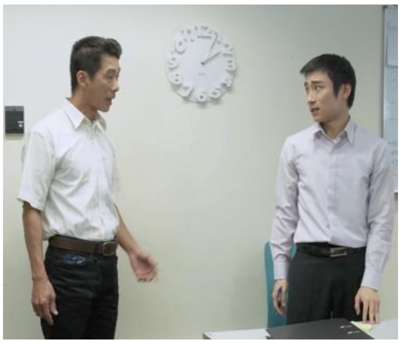

c) Version 3 was shown to younger male participants

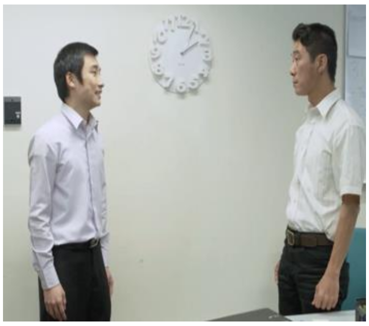

d) Version 4 was shown to older male participants

Note. For each version, the actor/actress ***on the right of the picture represents the main actor*** whereas the actor/actress on the left represents the coworker. The two actresses in Versions 1 and 2 were the same, and they swapped their role as either the main actor or coworker to match the age group of the target female participants. Similarly, the two actors in Versions 3 and 4 were the same, and they swapped their role as either the main actor or coworker to match the age group of the target male participants.

A sample video of intergenerational and non-intergenerational conflicts with English subtitles (with young female as the main actor) is available at the following websites (password: **cityu3442**)

An intergenerational conflict: https://vimeo.com/430216627

A non-intergenerational conflict: https://vimeo.com/430216825
